# Bidirectional regulatory mechanisms and therapeutic prospects of tumor hypercoagulable state and immunosuppressive tumor microenvironment

**DOI:** 10.3389/or.2026.1863533

**Published:** 2026-06-22

**Authors:** Yongli Lai, Ruyu Qin, Zhaidong Liu

**Affiliations:** 1 The First Clinical Medical College, Shandong University of Traditional Chinese Medicine, Jinan, China; 2 School of Traditional Chinese Medicine, Shandong Second Medical University, Weifang, China; 3 Department of Oncology, Affiliated Hospital of Shandong University of Traditional Chinese Medicine, Jinan, China

**Keywords:** anticoagulant therapy, bidirectional regulatory mechanism, cancer-associated thrombosis, immunosuppressive tumour microenvironment, immunotherapy, tumour hypercoagulable state

## Abstract

The tumor hypercoagulable state (THS) is an acquired and persistent prothrombotic condition driven by the tumor and its microenvironment. An immunosuppressive tumor microenvironment (TME) is a local microenvironment that suppresses antitumor immune responses and promotes tumor evasion of immune surveillance and is also a key driver of tumorigenesis, tumor progression, and metastasis. An intricate bidirectional regulatory axis exists between THS and immunosuppressive TME, establishing a self-reinforcing “hypercoagulability-immunosuppression” vicious cycle that severely impairs patient prognosis and reduces the efficacy of immunotherapy. To break this vicious cycle, the combined use of anticoagulant therapy and immunotherapy has garnered increasing attention. Current treatment techniques aimed at the coagulation-immune axis hold significant promise. However, this combinatorial method has a number of translational issues, including bleeding hazards, tumor heterogeneity, a lack of prognostic biomarkers, unexplained resistance mechanisms, and difficulties in clinical implementation. This review delineates the bidirectional regulatory mechanisms that link THS and immunosuppressive TME, assesses the promise of combined therapeutic modalities, discusses current challenges, and outlines future directions for precision oncology, with the ultimate goal of providing more effective cancer treatment options.

## 1 Introduction

Tumor hypercoagulable state (THS) is a pathophysiological modification in the hematological system of patients with malignant tumors that causes an enhanced tendency for blood coagulation as a result of the tumor and related treatments. Its pathophysiology is complex, including multiple synergistic pathways. First, tumor cells can directly express procoagulant factors or activate platelets and the coagulation cascade by releasing procoagulant microparticles and inflammatory cytokines, respectively. Second, systemic inflammatory responses caused by tumors, vascular endothelial injury, and treatments like chemotherapy and surgery enhance coagulation system activation (1). Finally, tumors can inhibit the protein C route and activate the fibrinolytic system, resulting in a relative lack of natural anticoagulant and fibrinolytic properties (2). THS’s principal clinical manifestation is cancer-associated thrombosis (CAT), with venous thromboembolism being the most common form and the second-highest cause of death in cancer patients (1).

The tumor microenvironment (TME) is the internal environment in which tumor cells develop and thrive. One of its major functional features is the creation of an immunosuppressive TME, a complex regulatory network that includes immunosuppressive cells, inhibitory cytokines and chemokines, immunosuppressive metabolites, immunological checkpoint molecules, and the extracellular matrix (3). Immunosuppressive cells function by depleting nutrients, making direct cells contact, and secreting inhibitory substances. Inhibitory cytokines and chemokines directly reduce effector T cells (Teffs), stimulate regulatory T cell (Treg) differentiation, and recruit more immunosuppressive cells. Lactate, glutamine (Gln), and amino acids suppress immunity by altering the physicochemical properties of the TME or inducing apoptosis in immune cells, whereas immune checkpoint molecules such as programmed death-ligand 1 (PD-L1) and cytotoxic T-lymphocyte-associated protein 4 (CTLA-4) contribute to immune cell exhaustion. Furthermore, cancer-associated fibroblasts release extracellular matrix components that create physical obstacles to Teff penetration (4). These components work together to orchestrate tumor immune evasion and promote tumor development.

Notably, clinical observations show that patients with THS are more likely to acquire resistance to immunotherapy. Patients who get immunotherapy, on the other hand, are more likely to develop CAT (5, 6). This points to a potential bidirectional regulatory link between THS and immunosuppressive TME. More importantly, the link between hypercoagulability and immunosuppression varies significantly across tumor types. Tumors with significant procoagulant activity, such as glioblastoma and pancreatic ductal adenocarcinoma, have not only extraordinarily high CAT rates but are also known to be innately resistant to immune checkpoint inhibitors (ICIs) (7). In contrast, melanoma patients have relatively low baseline coagulation activation, yet the clinical benefit signals from anticoagulant medication paired with ICIs are extremely substantial (8, 9). This co-occurrence pattern of a “hypercoagulable phenotype—ICIs resistance” and a “hypocoagulable phenotype—ICIs sensitivity” is far from coincidence, emphasizing the critical necessity for tumor type-specific tailored combination methods rather than a one-size-fits-all strategy.

However, previous research has generally focused on unidirectional mechanisms—that is, how THS affects immune function or how immunological dysregulation affects coagulation function. A rigorous examination of the dynamic, bidirectional regulatory network produced by their interplay, as well as the molecular distinctions between tumor types, is still absent. Precision treatment options focused on this interrelated axis remain limited. This review seeks to elucidate the bidirectional regulatory mechanisms between THS and immunosuppressive TME, as well as to investigate the potential and challenges of existing therapeutic strategies, with the goal of providing a theoretical foundation for the development of novel targeted therapies and personalized combination regimens.

## 2 Literature search

This study adopts a narrative review approach. A literature search was conducted using a predefined strategy. The electronic databases PubMed and Web of Science were searched for relevant papers from their inception to May 2026. The search strategy combined Medical Subject Headings terms and keywords, including but not limited to “tumor hypercoagulable state,” “cancer-associated thrombosis,” “immunosuppressive tumor microenvironment,” “immunotherapy,” “anticoagulant therapy,” and “mechanism.” Boolean operators (AND, OR) were used to refine the search.

Inclusion criteria comprised original basic or clinical research examining the relationships between malignancies, coagulation, and immunity, as well as high-quality reviews, meta-analyses, and relevant perspective papers. Conference abstracts, editorials without original data, and studies with insufficient methodological information were excluded.

## 3 Mechanisms by which THS modulates immunosuppressive TME

### 3.1 Procoagulant factors influence immune cell activity

THS is jointly regulated by multiple procoagulant factors. These include tissue factor (TF), coagulation factors, inflammatory cytokines, vascular endothelial growth factor (VEGF), platelet-derived growth factor (PDGF), and coagulation-related proteins (10). These molecules act not only as primary mediators of coagulation activation but also as critical links between the coagulation system and immunosuppressive TME.

#### 3.1.1 Regulatory effects of TF on immune cells

TF is the most important initiator of THS and a key regulator of the tumor immune milieu; it has strong immunomodulatory properties and mediates tumor immune evasion via many mechanisms. In terms of macrophage control, tumor-associated macrophages (TAMs) are classified as M1-TAMs or M2-TAMs, with M1-TAMs reducing tumor cells proliferation and progression and M2-TAMs promoting tumor advancement (11). TF can inhibit the polarization of macrophages toward the M1 phenotype via the mechanistic target of rapamycin complex 2/extracellular signal-regulated kinase (ERK)/protein kinase B (AKT) signaling pathway, lowering their phagocytic activity and tumor-killing potential (12). TF can also bind to coagulation factor VII/activated factor VII (FVII/FVIIa) on the surface of macrophages to form complexes, thus enhancing macrophage adhesion to the extracellular matrix, activating cytoskeletal reorganization associated with migration, and promoting macrophage recruitment to sites of inflammation or tumor tissue (13–15). The TF-FVIIa complex can also upregulate CD47 and stanniocalcin 1 on tumor cell surfaces via the protease-activated receptor 2 (PAR2)-microRNA-221/signal transducer and activator of transcription 3 (STAT3) axis, continually reducing macrophage phagocytosis and favoring immunological escape (16). In terms of regulating myeloid-derived suppressor cells (MDSCs), TF binds FVII and coagulation factor X (FX) to generate thrombin (FIIa), which then cleaves complement C5 to produce C5a. C5a binds to C5aR on the surface of MDSCs, mediating the recruitment of MDSCs to the tumor site. This leads to the formation of an immune-excluded microenvironment, thereby facilitating tumor immune escape (17). MDSC recruitment undermines immunotherapy efficacy by preventing ICIs from successfully reversing T cells exhaustion, reducing therapeutic results (18). Regarding neutrophil regulation, tumor-associated neutrophils (TANs) are categorized as N1-TANs or N2-TANs. The mechanism by which TF signaling directly regulates TAN polarization is unknown; however, the coagulation cascade initiated by TF can influence neutrophil recruitment and functional status via complement activation and chemokine release, implying that TF signaling may be involved in inducing TAN polarization towards the immunosuppressive N2 phenotype (19, 20). N2-TANs recruit Tregs while suppressing T cells and natural killer cells (NK cells) through molecules such as fatty acid transporter 2 and arginase-1, decreasing immune surveillance (21). They also regulate PD-L1 expression in both their own and tumor cells via interleukin 17 (IL-17) signaling, which reinforces immunosuppressive TME and reduces tumor susceptibility to programmed cell death protein 1 (PD-1)/PD-L1 inhibitors (22).

#### 3.1.2 Regulatory role of coagulation factors in immunosuppressive TME

Coagulation factors predominantly function through FVII, FX, prothrombin, and fibrinogen, regulating the TME via numerous mechanisms, with FIIa acting as the major hub of immunoregulation. FVII binds specifically to TF, forming the TF-FVIIa complex, which activates FX and converts prothrombin into FIIa. FIIa then causes tumor cells and stromal cells to produce immunosuppressive factors via protease-activated receptor 1 (PAR1) and PAR2 signaling, suppressing the cytotoxicity of T cells and NK cells and reducing the secretion of antitumor cytokines (7, 23). After activating PAR1, FIIa promotes the secretion of monocyte chemotactic protein-1 (MCP-1) by stromal and tumor cells via the phosphatidylinositol 3-kinase (PI3K)/AKT and mitogen-activated protein kinase kinase (MEK)/ERK1/2 signaling pathways (24, 25). MCP-1 specifically recruits circulating monocytes to the TME (26) and promotes their differentiation into M2 macrophages, thereby increasing immunosuppression (25, 27). Furthermore, FIIa can activate neutrophils via PAR1/4, resulting in the production of neutrophil extracellular traps (NETs) (28); NETs can trap tumor cells to form a protective barrier while degrading immune cell chemokines, hindering immune cell infiltration (29). FIIa also converts fibrinogen into fibrin, which deposits in the tumor stroma to form a mesh-like scaffold (30, 31), protecting MDSCs and TAMs from attack (32). It can also inhibit the infiltration of NK cells into the tumor core (33), reducing the efficacy of the antitumor immune response.

#### 3.1.3 Immunoregulatory functions of inflammatory cytokines

Inflammatory cytokines are small proteins secreted by immune cells, tumor cells, and stromal cells; among them, interleukin 6 (IL-6) and interleukin 10 (IL-10) are key regulators of immunosuppressive TME. IL-6 exerts bidirectional regulatory effects through two signaling modes. The first is the classical pathway, which binds to the membrane-bound IL-6 receptor and mediates anti-inflammatory and tissue repair effects. The second is the trans-signaling pathway, which binds to soluble IL-6R and activates gp130 that is widely expressed on various cell types, thereby driving pro-inflammatory and immunosuppressive effects (34, 35). Under acute inflammation or at low levels, IL-6 can activate the immune system. IL-6 stimulates the production of adhesion molecules and high endothelial venule adhesion via the STAT3 signaling pathway, boosting T cell migration to areas of inflammation (36, 37). Concurrently, IL-6 inhibits T cells death by increasing the anti-apoptotic genes B-cell lymphoma 2 and B-cell lymphoma-extra large. After T cell receptor activation, IL-6 acts as a costimulatory factor to promote the proliferation of CD4^+^ T cells and CD8^+^ T cells (38, 39). However, in the chronically inflamed TME, tumor cells and stromal cells constantly release high levels of IL-6, causing its immunosuppressive effects to predominate. First, IL-6 changes bone marrow hematopoiesis by stimulating STAT3 signaling, which causes myeloid precursor cells to develop into MDSCs (40). It also downregulates major histocompatibility complex class II (MHC-II) expression on MDSCs, thereby reducing their antigen-presenting capacity to T cells and hindering the initiation of antitumor immune responses (41, 42). Second, IL-6 suppresses T cell proliferation and T cell receptor expression by depleting L-arginine within TME (43), while also producing NO, which affects T cell function (44). Additionally, IL-6 directly increases PD-L1 expression on MDSCs surfaces, whereas interaction with PD-1 on T cells causes T cell dysfunction (45). IL-10 is another crucial inflammatory cytokine. First, it inhibits dendritic cells (DCs) maturation (46) and downregulates the expression of MHC-I/II molecules and co-stimulatory molecules on macrophages and DCs, reducing antigen presentation efficiency and impeding T cell activation (47, 48). Second, it directly downregulates the proliferative activity of CD4^+^ and CD8^+^ T cells, inhibiting their cytotoxic effects on tumor cells (49). Furthermore, it decreases interferon-gamma (IFN-γ) production by NK cells, which reduces their ability to detect and kill tumor cells (50). Finally, it induces proliferation of Tregs, enhancing immunosuppression (51).

#### 3.1.4 Immunoregulatory effects of VEGF

VEGF is not only a pro-angiogenic factor but also a significant immunoregulator. In terms of DCs regulation, VEGF can inhibit NF-κB activation via binding to vascular endothelial growth factor receptor 1 (VEGFR-1) on the surface of hematopoietic progenitor cells, thereby blocking DCs maturation at an early stage. The resulting reduction in mature DCs numbers impairs their ability to effectively present tumor antigens to and activate T cells (52). VEGF further induces high surface expression of PD-L1 on mature DCs. Binding of PD-L1 to PD-1 on T cell surfaces directly suppresses T cell cytotoxicity (53). Regarding MDSCs regulation, VEGF can induce tumor cells and stromal cells to secrete chemokines such as stromal cell-derived factor 1 via the VEGFR-2 signaling pathway, promoting immature myeloid cells chemotaxis and aggregation while preventing their differentiation into functional immune cells such as DCs (54). These effects are achieved through persistent Janus kinase (JAK)/STAT3 activation, which keeps MDSCs inhibitory and undifferentiated (55). Regarding immune cells infiltration regulation, VEGF-induced tumor vasculature exhibits structural abnormalities and heightened permeability (56). Loosened intercellular junctions in vascular endothelial cells permit plasma protein extravasation, creating interstitial tumor hypertension that impedes cytotoxic T lymphocytes (CTLs) and NK cell penetration into tumor core regions (57). VEGF-mediated vascular abnormalities also interfere with immune cells-endothelial cells interactions by downregulating the adhesion molecules intercellular adhesion molecule 1 and vascular cells adhesion molecule 1, preventing immune cells infiltration (58).

#### 3.1.5 Immunoregulatory effects of PDGF

PDGF is a key growth factor that regulates cells growth and division; it is primarily secreted by platelets and tumor cells and mediates tumor immune evasion through multiple pathways. In terms of NK cells regulation, platelet-derived PDGF can bind to platelet-derived growth factor receptor (PDGFR) on the surface of stromal or tumor cells, causing transforming growth factor-beta 1 (TGF-β1) to be released. By blocking mammalian target of rapamycin activity, TGF-β1 then downregulates the expression of natural killer group 2 member D, a crucial activation receptor on the surface of NK cells, which decreases the capacity of NK cells to identify and eliminate tumor cells (59, 60). In terms of TAM regulation, PDGF binds to PDGFR on the surface of TAMs to activate the IL-6/STAT3 signaling pathway and promote M2-TAMs (61). This accelerates tumor invasion and epithelial-mesenchymal transition (62, 63), which facilitates tumor cells escape. In terms of CTLs regulation, PDGF inhibits CTLs cytotoxicity by inducing TGF-β1 release (64, 65) while simultaneously reducing production of key cytokines such as interleukin 2 (IL-2), weakening the antitumor adaptive immune response (66).

#### 3.1.6 Protein S and Gas6: immunoregulatory functions of coagulation-related proteins

Protein S and growth arrest-specific protein 6 (Gas6) are two important coagulation-related proteins that play unique roles in bridging the coagulation system and immune regulation. Protein S not only serves as a cofactor in the protein C anticoagulant pathway but also acts as a bridging molecule that promotes the clearance of apoptotic cells. It binds to phosphatidylserine exposed on the surface of apoptotic cells while interacting with the MER proto-oncogene tyrosine kinase (MerTK) receptor on macrophages, activating downstream signaling and promoting macrophage differentiation into M2-TAMs. This process clears apoptotic cells while maintaining an immunosuppressive TME (67–69). Gas6 also acts as a bridging molecule that interacts with phosphatidylserine, mediating phagocytosis by activating the Axl and MerTK receptors. In immunosuppressive TME, the Gas6-Axl signaling pathway suppresses pro-inflammatory cytokine release, promotes DC acquisition of an immunotolerant phenotype; at the same time, it enhances M2-TAM differentiation via the PI3K/AKT and MEK/ERK pathways (70, 71), thus reinforcing immunosuppressive effects.

In summary, tumor-associated procoagulant factors (including TF, coagulation factors, inflammatory cytokines, VEGF, PDGF, protein S/Gas6, etc.) collectively shape an immunosuppressive TME through multifaceted, synergistic mechanisms. As shown in Table 1, these mechanisms can be categorized into three core levels—functional, cellular, and structural—which work in concert to drive the development of immune evasion.

**TABLE 1:** Mechanisms by which procoagulant factors maintain an immunosuppressive TME.

Coagulation factors/Molecules	Target cells	Mechanism	Result	References
TF	Macrophage	Inhibition of M1 polarization via the mTORC2/ERK/AKT pathway	Reduces the phagocytic activity and tumour-killing capacity of macrophages, thereby promoting immune evasion	(11, 12)
		The TF-FVIIa complex enhances the adhesion of macrophages to the ECM and promotes their recruitment		(13–15)
		TF-FVIIa upregulates CD47/STC1 via the PAR2-miR221/STAT3 axis, thereby inhibiting phagocytic function		(16)
	MDSCs	TF activates thrombin in conjunction with FVII and FX → the cleavage of complement C5 produces C5a →C5a binds to C5aR on the surface of MDSCs	Mediates the local accumulation of MDSCs at the tumour site, creating an immunosuppressive microenvironment and reducing the efficacy of ICIs	(17, 18)
	Neutrophils	TF initiates the coagulation cascade → activation of complement and chemokines→ induce neutrophil N2-type polarization TAN type N2 inhibits T/NK cells via FATP2 and Arg-1 and upregulates PD-L1	Enhanced immunosuppression	(19–22)
Coagulation factors (FVIIa, FXa, thrombin, fibrin)	T cells, NK cells	FIIa induces the release of immunosuppressive factors from tumor and stromal cells via PAR1/PAR2 signaling	Reduce the secretion of antitumor cytokines	(7, 23)
	Monocytes	Thrombin activates PAR1 → PI3K-Akt,MEK-Erk1/2 → increased MCP-1 secretion↑ → recruit monocytes and induce M2 macrophage differentiation	Enhance immunosuppression	(24–27)
	Neutrophils	Thrombin activates neutrophils via PAR1/4 → NETs release	Inhibits the infiltration of immune cells	(28, 29)
	MDSCs, TAMs	FIIA converts FI into Fbn, forming a fibrin scaffold that protects MDSCs/TAMs from NK-mediated clearance and inhibits NK infiltration	Reduce the efficacy of the antitumor immune response	(30–33)
IL-6	MDSC	Downregulation of MHC-II; Increase PD-L1	MDSCs accumulate and suppress T cells	(40–42, 45)
	T cells	Consumption of L-arginine,Produce NO	Inhibition of T-cell proliferation and function	(43, 44)
IL-10	DC/macrophage	Inhibiting DC maturation; Downregulation of macrophages,MHC and co-stimulatory molecules	Impairment of antigen presentation hinders T-cell activation	(46–48)
	CTL/NK	Directly reduces the proliferative activity of CTLs such as CD4^+^ and CD8^+^ cells and inhibits IFN-γ production	Decreased cytotoxic activity	(49, 50)
	Treg	Induce Treg proliferation	Enhance immunosuppression	(51)
VEGF	DC	Binding to VEGFR-1 →Inhibit NF-κB activation→Inhibit DC maturation; Induce PD-L1 expression in mature DCs	Inhibition of T-cell cytotoxicity	(52, 53)
	MDSCs	Via VEGFR-2 →Induce tumor cells and stromal cells to secrete SDF-1/CXCL12→ Isolation of immature myeloid cells	Accumulation of MDSCs, Suppress the immune response	(54)
​	CTL,NK cells	Induce abnormal blood vessels; Downregulation of the adhesion molecules ICAM-1 and VCAM-1	Inhibits the infiltration of immune cells	(56–58)
PDGF	NK cells	Induce the release of TGF-β1→Inhibition of mTOR → downregulation of NKG2D expression	Reduce the ability of NK cells to recognize and kill tumor cells	(59, 60)
	TAMs	In conjunction with PDGFR→Activate the IL-6/STAT3 pathway→Promote M2-type polarization	Promotes tumor invasion and epithelial-mesenchymal transition	(61–63)
	CTL	Induce the release of TGF-β1→Inhibition of CTL cytotoxicity; reduce IL-2 production	Weakening the adaptive antitumor immune response	(64–66)
Protein S	Macrophage	Acts as a bridging molecule that binds to phosphatidylserine on the surface of apoptotic cells and interacts with the MerTK receptor	Maintain an immunosuppressive microenvironment	(67–69)
Gas6	Macrophages, DC	In conjunction with polarization, activating Axl/MerTK; Enhance M2 polarization via the PI3K-AKT and MEK-ERK pathways; Inhibit the release of pro-inflammatory factors	Enhance immunosuppression	(70, 71)

### 3.2 THS leads to hypoxia, exacerbating immunosuppression

Tumor cells produce TF, procoagulants, and other compounds that activate the coagulation cascade, resulting in platelet aggregation and fibrin deposition. This creates microthrombi within tumor microvessels, blocking local blood flow and causing perfusion insufficiency and hypoxia (72). Hypoxia exacerbates immunosuppression through a variety of mechanisms. First, hypoxia can impair the activity of effector immune cells. Hypoxia inhibits CD8^+^ T cell proliferation and differentiation by reducing IFN-γ and IL-2 production via hypoxia-inducible factor 1 (HIF-1)-dependent pathways (73). Hypoxia can also stimulate autophagy in tumor cells, destroy granzyme B produced by NK cells, and prevent NK cell-mediated tumor cell death (74). At the same time, it modulates the shedding of the MICA molecule on tumor cells surfaces via HIF-1, lowering NK cell activation signals and cytotoxicity (75). Second, hypoxia promotes the recruitment of immunosuppressive cells. Hypoxia upregulates arginase activity and NO production in MDSCs via HIF-1, thereby enhancing their inhibitory effects on T cells (76). Hypoxia also induces tumor cells and stromal cells to secrete TGF-β via HIF-1. In the presence of TGF-β, HIF-1 can directly bind to the Foxp3 promoter, inducing Treg formation (77). Hypoxia causes tumor cells to produce CCL28 and TGF-β1, which attract Tregs to infiltrate the TME (78), increasing immune suppression. Finally, hypoxia can upregulate the expression of immune checkpoint molecules. Hypoxia significantly upregulates PD-L1 expression on the surface of tumor cells by directly binding to the PD-L1 promoter via HIF-1, thereby inhibiting T cells activation (79). It also increases the expression of PD-L1/PD-L2 on endothelial cells surfaces, which bind to PD-1 on T cells, restricting T cell penetration into tumor tissue and aggravating immune evasion (80). Local tissue hypoxia caused by THS not only compromises the function and survival of effector immune cells, but it also encourages the recruitment and activation of immunosuppressive cells. Furthermore, it widely upregulates immune checkpoint molecules like PD-L1, aggravating immunosuppressive TME on numerous levels, including cell function, cell composition, and immune regulatory signaling.

### 3.3 THS promotes the formation of physical barriers, impeding immune cells infiltration

THS can cause fibrin development, resulting in a physical barrier that blocks immune cells from entering the tumor core. Fibrin develops sheath-like structures around tumor vessels, preventing peripheral blood immune cells from making direct contact with tumor cells (81). It also collaborates with NETs to produce denser fibrin-NET complexes on tumor vascular endothelial cells, thereby increasing the physical barrier effect. This complex not only blocks CD8^+^ T cell migration but also degrades the immunostimulatory chemokine C-X-C motif chemokine ligand 9, reducing NK cells recruitment (82). Furthermore, fibrin-platelet complexes create a more stable barrier on tumor vascular endothelial surfaces. This barrier not only physically obstructs NK cells migration but also further suppresses NK cells activity through TGF-β1 released by platelets (33). The barrier formed by THS not only directly impedes the infiltration of effector immune cells into the tumor parenchyma but also further impairs immune cells recruitment and function through mechanisms such as degrading chemokines and releasing inhibitory factors, thereby consolidating immunosuppressive TME.

In summary, THS drives immunosuppressive TME through three synergistic levels: at the molecular level, procoagulant factors directly suppress effector immune cells function while inducing the recruitment of inhibitory cells; at the metabolic level, tumor hypercoagulation induces hypoxia, which broadly upregulates PD-L1 via the HIF-1α pathway and expands immunosuppressive cells populations; at the structural level, fibrin forms physical barriers with platelets and NETs, blocking immune cells infiltration. Through the synergistic effects of signaling inhibition, hypoxic metabolism, and physical barriers, THS comprehensively suppresses antitumor immunity functionally, metabolically, and spatially.

## 4 Mechanisms by which immunosuppressive TME promotes THS

### 4.1 Immunosuppressive cells activate the coagulation pathway

#### 4.1.1 MDSCs

MDSCs constitute a population of immature bone marrow cells and serve as core immune cells promoting THS. Under immunosuppressive TME induction, MDSCs stably express TF and disseminate procoagulant signals by releasing TF-containing extracellular vesicles (83). As the primary physiological initiator of the extrinsic coagulation pathway, TF forms a complex with FVII to activate FX, thereby initiating the extrinsic coagulation cascade and ultimately leading to fibrin deposition (84). Moreover, the P-selectin glycoprotein ligand-1 on the surface of MDSCs binds to P-selectin on platelets, promoting platelet activation and aggregation (85). MDSCs can also indirectly induce neutrophils to release NETs by secreting ROS (86). The histones and neutrophil elastase within NETs can damage vascular endothelial cells, thereby activating platelets and FXII and amplifying the coagulation response (87).

#### 4.1.2 M2-TAMs

TAMs represent another core cell type directly involved in coagulation activation within immunosuppressive TME. On one hand, M2-TAMs can autonomously synthesize coagulation factors and initiate the extrinsic coagulation pathway. TAMs themselves synthesize and express TF, FVII, and FX, forming a TF-FVIIa-FXa activation complex locally. Through the FXa/PAR2 cell-autonomous signaling pathway, this complex positively upregulates the expression of TF, FVII, and FX by the macrophages themselves, initiating and amplifying the extravascular coagulation cascade locally without relying on the circulating coagulation system (88). Concurrently, the binding of FXa generated by the coagulation cascade to PAR2 on the TAM surface maintains the immunosuppressive polarized phenotype of TAMs and promotes their secretion of pro-angiogenic factors and immunosuppressive cytokines, thereby tightly coupling local coagulation activation with immunosuppressive TME (89). On the other hand, M2-TAMs can activate platelets and amplify the intrinsic coagulation pathway via the ligand Gas6. TAMs upregulate prostaglandin E synthase expression through the Gas6-activated ERK1/2 signaling pathway, promoting the generation and release of prostaglandin E_2_ (PGE_2_). PGE_2_ activates platelets and releases procoagulant substances like polyphosphates, amplifying the intrinsic coagulation cascade (90).

#### 4.1.3 Tregs

As key regulatory cells in immune tolerance, Tregs indirectly stabilize the procoagulant environment by suppressing inflammatory responses and maintaining immune tolerance. First, Tregs exert their effects through cell-contact-dependent mechanisms; CTLA-4, which is highly expressed on their surface, specifically binds to the co-stimulatory molecules CD80/CD86 on antigen-presenting cells, competitively blocking activation signals to Teffs while simultaneously inducing downregulation of antigen-presenting cells function. This inhibits damage to the vascular endothelium caused by excessive inflammatory responses, maintains local vascular integrity, and provides a stable platform for the sustained progression of the coagulation cascade (91, 92). Second, Tregs release key inhibitory cytokines such as IL-10 and TGF-β via paracrine pathways. These cytokines suppress the pro-inflammatory activity of macrophages, reduce the production of pro-inflammatory factors such as tumor necrosis factor alpha (TNF-α) and interleukin 1 beta (IL-1β), and decrease inflammation-mediated inactivation of TF (93–95). Through these mechanisms, Tregs effectively suppress excessive inflammatory responses in immunosuppressive TME, thereby indirectly maintaining the stability of THS.

### 4.2 Immunosuppressive cytokines drive coagulation system dysregulation

Immunosuppressive cytokines that are abundant in immunosuppressive TME serve as key mediators, promoting both tumor immune evasion and a dysregulated coagulation-anticoagulation system. They contribute to THS by influencing thrombopoiesis, coagulation factor activity, and the fibrinolytic system.

#### 4.2.1 TGF-β: the upstream core regulator of the coagulation-anticoagulation balance

TGF-β is one of the most abundant immunosuppressive cytokines in immunosuppressive TME, and its sources are diverse, including tumor cells, Tregs, and platelets. Notably, platelets serve as an important reservoir of TGF-β in the TME; latent TGF-β is anchored to the platelet surface via its binding to glycoprotein A repetitions predominant and can be directly cleaved and activated by FIIa, releasing bioactive TGF-β (96). Activated TGF-β binds to the TGF-β receptor I/II (TβRI/II) complex on the tumor cells surface, activating the SMAD family member 2/3 signaling cascade and phosphorylating ERK1/2 to activate the transcription factor Activator Protein-1 (97). These two pathways converge at the TF gene’s promoter region, increasing TF transcriptional expression and effectively initiating the extrinsic coagulation pathway (98, 99). At the same time, TGF-β signaling inhibits the expression of thrombomodulin, thereby attenuating the thrombomodulin-dependent protein C anticoagulation pathway (100).

#### 4.2.2 IL-6: regulating platelet production and coagulation factor activity

IL-6 is not only a key immunosuppressive cytokine in immunosuppressive TME but also an independent risk factor for tumor-associated hypercoagulability and thrombosis. Its core regulatory mechanisms center on promoting platelet production and enhancing coagulation factor activity (101, 102). In terms of platelet production regulation, IL-6 operates through two primary pathways. On the one hand, via the IL-6-thrombopoietin-megakaryocyte-platelet axis, it upregulates thrombopoietin through the JAK/STAT3 pathway and synergizes with related cytokines to promote megakaryocyte maturation and platelet release, thereby enriching highly reactive immature platelets (103, 104). On the other hand, it pre-activates platelets through soluble IL-6R/gp130 trans-signaling, enhancing their reactivity and aggregability (105, 106). With respect to coagulation factor activity regulation, IL-6 can induce high expression of TF and upregulate the synthesis and activity of coagulation factors and fibrinogen, activating both the intrinsic and extrinsic coagulation pathways (107). Additionally, it potently induces plasminogen activator inhibitor-1 (PAI-1) while downregulating tissue-type plasminogen activator (tPA), suppressing the fibrinolytic system (108).

#### 4.2.3 IL-10: an endogenous regulator of coagulation and fibrinolysis balance

IL-10, a potent anti-inflammatory and immunomodulatory cytokine, promotes THS via cell-specific mechanisms that inhibit fibrinolysis and increase platelet activity. IL-10 inhibits fibrinolysis via activating the PI3K-AKT pathway, which promotes PAI-1 transcription and expression. PAI-1 forms irreversible complexes with tPA and urokinase-type plasminogen activator (uPA), inhibiting their fibrinolytic action, decreasing plasmin production, and causing fibrin deposition (109, 110). In terms of platelet activation, IL-10 binds to the IL-10R on the platelet surface, activating the downstream STAT3 signaling pathway (111), increasing the activity of platelet surface GPIIb/IIIa receptors, and boosting platelet aggregation (112, 113).

**FIGURE 1:**
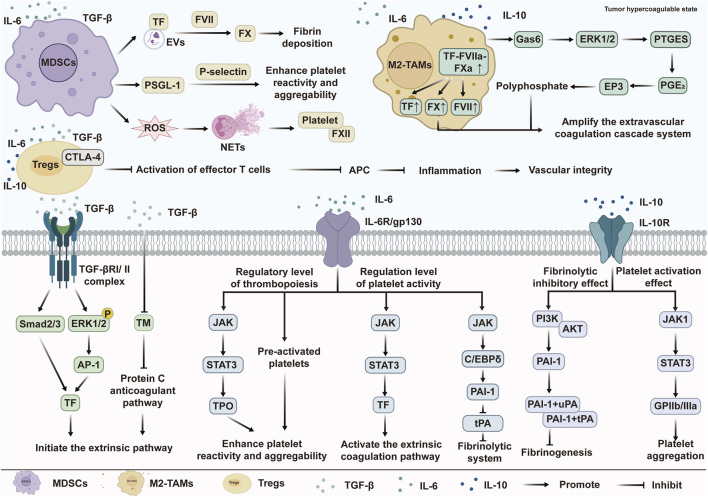
Mechanisms of immunosuppressive cells and cytokines in regulating THS. In the figure, purple cells represent MDSCs, light beige cells represent M2-TAMs, and light yellow cells represent Tregs; the light gray, green, and dark blue scatter plots represent TGF-β, IL-6, and IL-10, respectively; → indicates promotion/activation, and ⊣ indicates inhibition/blockade.

Immunosuppressive cells and factors in immunosuppressive TME work together to impact THS via multilevel synergistic pathways. As depicted in Figure 1, these mechanisms are specifically as follows: Immunosuppressive cells stimulate coagulation-related factors and protect them from immune cell-mediated degradation; immunosuppressive cytokines regulate the balance of the coagulation system via multiple signaling pathways.

### 4.3 Dysregulation in immunosuppressive TME

The establishment of immunosuppressive TME is intimately associated with metabolic dysregulation. Tumor cells use metabolic reprogramming to change the distribution of nutrients and release immunosuppressive chemicals, all while participating in metabolic competition and mutual regulation with immune and stromal cells (114). This ultimately reduces the activity of effector immune cells, promotes the buildup of immunosuppressive cells, and exacerbates THS.

#### 4.3.1 Procoagulant effects of lactate

In immunosuppressive TME, both tumor and immunosuppressive cells exhibit the characteristic of enhanced aerobic glycolysis, as evidenced by high expression of glucose transporters and key glycolytic enzymes, resulting in competitive glucose consumption and massive lactate accumulation (115). Lactate promotes THS through multiple mechanisms. First, it reduces platelet intracellular pH, increasing their responsiveness to collagen and FIIa, which speeds up platelet aggregation and thrombus formation (116, 117). Second, lactate causes endothelial cells and tumor cells to overexpress PAI-1, which inhibits tPA and uPA This prevents plasmin production and inhibits fibrin breakdown (113, 118). Finally, lactate promotes the formation of NETs. The DNA-fiber network structure of NETs can directly adsorb platelets and red blood cells, bind to fibrinogen, and, under the action of FIIa, promote fibrin generation, leading to the formation of stable thrombi (119, 120).

#### 4.3.2 The coagulation-regulatory role of Gln

Gln addiction represents another core metabolic feature of immunosuppressive TME. Tumor cells consume Gln in the TME via the high expression of solute carrier family 1 member 5 and glutaminase 1 to support their rapid proliferation and immunosuppressive functions (121). Gln deficiency not only induces metabolic dysfunction in Teffs and DCs due to nutritional deprivation but also indirectly potentiates the procoagulant activity of immunosuppressive cells (122). Furthermore, Gln is catalyzed by glutaminase 1 to generate glutamate, which is subsequently converted into α-ketoglutarate to enter the tricarboxylic acid cycle. Ammonia produced during this process can inhibit the anticoagulant function of vascular endothelial cells (123, 124) and induce IL-6 secretion from M2-TAMs. IL-6 promotes thrombopoietin secretion and enhances FVIII activity through the JAK/STAT3 pathway (125, 126). α-ketoglutarate can also directly activate the extrinsic coagulation pathway by regulating KDM6A activity, reducing the inhibitory histone H3K27me3 modification, and upregulating TF expression in tumor cells (127–129). These mechanisms closely link Gln metabolism to THS.

#### 4.3.3 Procoagulant mechanisms of amino acid and adenosine

Beyond Gln, anomalies in amino acid metabolism, such as tryptophan and arginine, as well as adenosine accumulation, aggravate THS by modulating key molecules in immunosuppressive and coagulation pathways (114). Regarding tryptophan metabolism, MDSCs and M2-TAMs highly express IDO, which converts tryptophan into kynurenine. Kynurenine, on one hand, induces T cells apoptosis and promotes Treg proliferation by activating the activating the aryl hydrocarbon receptor (AHR) (130). On the other hand, it can upregulate TF expression on the surface of tumor cells and endothelial cells via the AHR-IL-6-STAT3 signaling axis, activating the extrinsic coagulation pathway (131, 132). Regarding arginine metabolism, MDSCs deplete arginine within the TME through high expression of arginase-1 and inducible nitric oxide synthase (iNOS) (133, 134). Concurrently, under specific conditions, iNOS-derived NO promotes platelet secretion and aggregation via the cyclic guanosine monophosphate pathway (135). Adenosine metabolism is another important regulatory axis. The CD39/CD73 enzyme complex, found on the surface of tumor cells and immunosuppressive cells, converts ATP into adenosine (136). Adenosine directly reduces Teff activity via the adenosine A_2_A receptor (A_2_AR) (137, 138), while also promoting platelet aggregation (139). Notably, during inflammation or hypoxia, adenosine can inhibit monocyte TF expression via the adenosine A_3_ receptor, thereby blocking the initiation of coagulation. It can also suppress the release of pro-inflammatory factors such as TNF-α and IL-12 via the A_2_AR reducing the inducers of TF and the triggers of the extrinsic coagulation pathway (140).

In summary, immunosuppressive TME regulates THS through a synergistic “cell-cytokine-metabolism” network. Immunosuppressive cells directly express procoagulants and activate platelets, thereby initiating the coagulation cascade; immunosuppressive cytokines systematically disrupt the coagulation-fibrinolysis balance; the products of metabolic reprogramming directly induce coagulation activation while suppressing the immune response. The combined effect of these three factors exacerbates THS.

## 5 Integration of bidirectional regulatory loops and cancer-specific driving patterns

As discussed earlier, THS suppresses antitumor immunity via three pathways: procoagulant factors, hypoxia, and physical barriers. This also elucidates how immunosuppressive cells and inhibitory cytokines, combined with metabolic disorders, reciprocally activate the coagulation cascade and aggravate cancer-associated hypercoagulability. The two regulatory processes are neither independent nor parallel, but form a mutually reinforcing and self-sustaining vicious cycle via multi-site crosstalk. To further elucidate this bidirectional regulatory axis, this section reviews unified regulatory networks, screens potential pharmacological intervention targets, and clarifies the dominant initiating events in distinct tumor subtypes, thus establishing a solid theoretical basis for precise targeted tumor therapy.

### 5.1 A unified loop model of the bidirectional vicious cycle between hypercoagulability and immunosuppression

Combining the mechanisms described in Sections 3, 4, the bidirectional regulation between THS and TME can be summarized as a self-reinforcing closed loop consisting of the following five sequential steps. Each of these five steps is supported by mechanistic evidence presented in Sections 3, 4; therefore, the specific molecular pathways will not be repeated here:

①Coagulation initiation: Tumor cells and immunosuppressive cells (MDSCs, M2-TAMs) express TF, release TF^+^ extracellular vesicles, and activate the extrinsic coagulation cascade (TF → FVIIa → FXa → FIIa → fibrin). At the same time, M2-TAM can autonomously synthesize FVII and FX, forming a TF-FVIIa-FXa activation complex at the tumor site to initiate extravascular coagulation independently of the circulatory system. ② Induction of immunosuppression: FIIa directly inhibits the cytotoxic activity of CD8^+^ T cells and NK cells via the PAR signaling pathway; fibrin and NETs form a physical barrier that blocks the infiltration of immune cells and degrades the chemokine CXCL9. At the same time, microthrombi induce hypoxia, which suppresses effector cells function via the HIF-1α pathway and upregulates PD-L1 expression. ③ Release of inhibitory factors and metabolic reprogramming: The process described above induces the expansion of MDSCs, M2-TAMs, Tregs, and other cells, leading to the secretion of TGF-β, IL-6, and IL-10. Concurrently, enhanced glycolysis results in lactate accumulation, Gln metabolism produces α-KG, and adenosine accumulates via the CD39/CD73 pathway, thereby creating an immunosuppressive metabolic microenvironment. ④ Positive feedback amplification in the coagulation system: Inhibitory factors and metabolic products amplify the coagulation system through positive feedback: TGF-β upregulates TF and inhibits the protein C pathway; IL-6 and IL-10 promote platelet production and activation and upregulate PAI-1 to inhibit fibrinolysis; lactate and α-ketoglutarate further promote platelet aggregation and TF expression. ⑤ Loop Steady-State Stabilization: Newly generated platelets and persistent fibrin-NET complexes further reinforce the physical barrier, blocking the infiltration of effector immune cells; metabolites provide a continuous energy supply, sustaining the immunosuppressive phenotype of MDSCs and M2-TAMs; PAI-1-mediated inhibition of fibrinolysis leads to persistent fibrin deposition. Step 5, in turn, further reinforces Step 1, creating a closed positive feedback loop (①→②→③→④→⑤→①).

Within this pathway, the following nodes show clear potential for drug intervention (see Section 6 for details): the TF/PAR1 axis, FXa/FIIa, platelets, IL-6/IL-6R, and C5a/C5aR, as well as lactate, Gln, and adenosine in the metabolic pathway.

### 5.2 Dominant initiating events in different tumor types

There are significant tumor-specific differences in the initiating steps of the hypercoagulable-immunosuppressive cycle. Based on baseline levels of coagulation activation, common tumors can be classified into two major categories: the hypercoagulable phenotype and the hypocoagulable phenotype.In hypercoagulable tumors such as glioblastoma and pancreatic ductal adenocarcinoma, the cycle often begins with a hypercoagulable state as the dominant initiating event—tumor cells overexpress TF and procoagulant microparticles, driving excessive activation of the coagulation cascade, which in turn shapes an immunosuppressive TME, ultimately leading to intrinsic resistance to ICIs therapy. In contrast, in tumors with a hypocoagulable phenotype, such as melanoma, this cycle may begin with immune suppression as the primary initiating event—the tumor establishes an immunosuppressive TME by recruiting MDSCs and Tregs, thereby indirectly activating the coagulation pathway. For this reason, anticoagulant therapy in tumors with a hypocoagulable phenotype can indirectly alleviate immunosuppression by breaking the “immunosuppression → hypercoagulability” feedback loop, thereby producing a more pronounced synergistic effect.

In summary, THS and immunosuppressive TME form a mutually reinforcing, self-perpetuating vicious cycle through the five-step closed-loop described above. There are significant differences in the targetability of different nodes within this circuit, and variations in the dominant initiating events across different tumor types provide a theoretical basis for personalized combination therapy.

## 6 Coagulation-immunomodulatory targeted therapies

As discussed earlier, the THS and immunosuppressive TME mutually drive each other through multiple signaling pathways, creating a self-reinforcing hypercoagulable-immunosuppressive vicious cycle that not only increases the risk of CAT but also significantly weakens the antitumor immune response and reduces the efficacy of immunotherapy. Therefore, the key strategy for breaking this vicious cycle should not be limited to thrombus prevention but should instead precisely target the key signaling nodes where the two interact.

### 6.1 Targeting TF and PAR receptors: precise intervention at upstream signaling nodes

TF is the upstream initiator of the coagulation-immune interaction, while PAR1/2 serves as the central hub for the conversion of procoagulant signals into immunosuppressive signals. Targeting these nodes holds promise for interrupting the vicious cycle at the earliest stage of signal transduction.

#### 6.1.1 Strategies for targeting TF: TF vaccines

As a key initiator of the coagulation-immune axis, TF has demonstrated significant potential for antitumor applications through targeted vaccines. The trivalent adjuvant vaccine KRN7000-TF·vizantin, developed by Yang et al, can simultaneously bind to CD1d and the Mincle receptor, synergistically activating iNKT cells and the Mincle signaling pathway, significantly enhancing the TF-specific IgG antibody response, and inducing a T helper 1 (Th1)/T helper 2 (Th2) mixed immune response dominated by Th1 cells. In a mouse model of breast cancer, the combination of this vaccine with cyclophosphamide significantly improved survival rates and prolonged median survival, confirming its ability to elicit a potent antitumor immune response (141).

#### 6.1.2 PAR1-targeted drug: vorapaxar

PAR1 and PAR2 serve as central signaling hubs linking coagulation activation, angiogenesis, and immune evasion. They primarily mediate downstream effects through the FIIa/PAR1 and TF-FVIIa/PAR2 pathways, making them key targets for combined anticoagulant and immunotherapeutic strategies (24, 142). Vorapaxar is a competitive PAR1 antagonist that exerts its effects by blocking FIIa-mediated platelet activation (143). Zhou et al. found in a mouse model of melanoma that vorapaxar directly targets the FOXO1/HMOX1 signaling axis, enhances the secretion of IFN-γ and granzyme B by tumor-infiltrating CD8^+^ T cells, and thereby significantly improves the efficacy of anti-PD-1 immunotherapy—particularly in “cold tumors” resistant to immunotherapy (144). It is worth noting that this immunomodulatory effect does not depend on the inhibition of platelet activation, suggesting that PAR1 antagonists possess immunomodulatory functions independent of their anticoagulant effects.

### 6.2 Targeting coagulation factors: blocking key effector molecules

Coagulation factors are not only the central effector molecules of the coagulation cascade, but also serve as a key signaling hub linking coagulation and immunosuppression. Drugs that target clotting factors can inhibit thrombosis while also remodeling immunosuppressive TME.

#### 6.2.1 Drugs that target FXa: rivaroxaban

Rivaroxaban, a commonly used direct oral anticoagulant, specifically binds to the active site of FXa, thereby blocking FXa-mediated immunosuppressive signaling (145). Graf et al. found in the MC38 colorectal cancer mouse model that rivaroxaban targets FXa synthesized by myeloid cells, reshapes the TME via the FXa/PAR2 signaling pathway, reduces Treg infiltration, increases the number of CD8^+^ T cells, and inhibits the polarization of macrophages toward the M2 phenotype. When used in combination with anti-PD-L1 therapy, there was a significant increase in CD137^+^ activated CD8^+^ T cells within the TME, indicating a synergistic effect between rivaroxaban and PD-1/PD-L1 inhibitors (146). A retrospective real-world study involving 280 patients with stage IV melanoma further supports this synergistic effect: the objective response rate (ORR) for rivaroxaban combined with ICIs reached 69.2%, compared with 36.4% for ICIs alone; median progression-free survival (PFS) for the combination therapy was 12 months, compared with 6.8 months for monotherapy (147). It is worth noting that Zimmermann et al. conducted a retrospective cohort analysis of 1,296 patients with *BRAF*-mutated advanced melanoma who received targeted therapy as part of the same ADOReg registry study. They found that patients receiving anticoagulant therapy, particularly FXa inhibitors, also demonstrated a significant survival benefit: The hazard ratio (HR) for 12-month PFS was 0.55, and the HR for overall survival (OS) was 0.35. This suggests that anticoagulant therapy may have a potential synergistic antitumor effect in melanoma patients receiving ICIs or targeted therapy (148).

#### 6.2.2 FIIa-targeted drugs: dabigatran etexilate

Dabigatran etexilate is a direct FIIa inhibitor that can exert synergistic antitumor effects when used in combination with chemotherapy and immunotherapy (149). Alexander et al. found in a mouse model of ovarian cancer that the combination of dabigatran etexilate and cisplatin reduced the infiltration of MDSCs and M2 macrophages and significantly increased IFN-γ secretion by CD8^+^ T cells in the ascites. When combined with anti-PD-1 antibodies, this further activated NK cells and reduced the tumor metastasis burden (150). The same team demonstrated in a breast cancer model that the combination of cyclophosphamide and dabigatran reverses tumor-induced splenomegaly, reduces the proportion of MDSCs in the spleen, and restores TGF-β levels in the spleen to normal (151). Metelli et al. demonstrated in MC38 colorectal cancer and EMT-6 breast cancer models that combination therapy with dabigatran etexilate and anti-PD-1 monoclonal antibodies significantly retarded tumor progression and prolonged survival in mice (152). A clinical study by Kött et al. further supports the benefits of combined anticoagulation and immunotherapy. They included 2,419 patients with advanced, unresectable melanoma treated with ICIs, 132 of whom received DOACs (including dabigatran) in combination. The results showed that the group receiving DOACs in combination with ICIs had significantly prolonged OS, with a 32% reduction in the risk of death and a median PFS of 11 months (153).

#### 6.2.3 Multitarget anticoagulants: heparins

Heparin-based drugs include low-molecular-weight heparin (LMWH) and unfractionated heparin (UFH). LMWH primarily inhibits FXa and has a weaker inhibitory effect on FIIa; UFH potently inhibits both FXa and FIIa simultaneously. In addition to its anticoagulant effects, heparin also exerts multifunctional immunomodulatory effects, including anti-inflammatory and vasoprotective actions (154). Quan et al. found in a mouse model of colorectal cancer that LMWH alone had no significant antitumor effect, but when combined with adoptive cells therapy or anti-PD-1 monoclonal antibodies, it significantly inhibited tumor growth and metastasis.Mechanistically, LMWH promotes tumor vascular normalization, alleviates local hypoxia, downregulates immune checkpoint expression, and upregulates the chemokine C-X-C motif chemokine ligand 10, ultimately enhancing CD8^+^ T cells infiltration and creating a more favorable microenvironment for immunotherapy (155). Wei et al. further validated this using immune desert (Panc02), immune exclusion (CT26), and T cells inflammation (MC38) models. The combination of LMWH with ACT or anti-PD-1 therapy can inhibit abnormal angiogenesis, reduce tumor stromal hypertension and THS, while simultaneously upregulating immune cell infiltration. It enhances the infiltration and activation of CD8^+^ T cells and M1 macrophages, and attenuates the immunosuppressive functions of Tregs and M2 macrophages, ultimately achieving antitumor and antimetastatic effects (156).

Although the above findings are encouraging, not all clinical results are consistent. In a clinical trial involving 728 patients with advanced cancer, Johannet et al. found that the combination of ICIs and therapeutic anticoagulation did not significantly improve key endpoints such as ORR, PFS, and OS; instead, it significantly increased the risk of bleeding (157). Nichetti et al. also reported in another study involving 217 patients with locally advanced or metastatic non-small cell lung cancer that the combination of anti-PD-L1 therapy and antiplatelet agents did not significantly improve PFS or OS (158). However, when interpreting these negative results, it is important to carefully consider the following three key factors.

First, selection bias inherent to observational studies cannot be ignored. In the studies conducted by Johannet and Nichetti, most patients administered anticoagulants received such treatment due to CAT, which itself serves as an independent marker of poor prognosis. In brief, these patients presented with more advanced disease and inherently worse clinical outcomes. Their unsatisfactory therapeutic responses may result from the high malignant aggressiveness of tumors, rather than the impairment of ICIs efficacy induced by anticoagulant administration. Second, the category discrepancy of anticoagulants is a core determinant influencing the efficacy of combined regimens. Rivaroxaban acts as a selective FXa inhibitor and directly remodels the tumor immune microenvironment via blocking the FXa/PAR2 signaling cascade. By contrast, LMWH exerts inhibitory effects on both FXa and FIIa, and possesses pleiotropic properties including anti-inflammatory activity and vascular permeability regulation; nevertheless, whether these additional functions participate in immune microenvironment remodeling remains poorly elucidated. Notably, the favorable clinical outcomes reported by Haist et al. and Kött et al. were mainly observed in patients receiving FXa inhibitors. In the Johannet cohort, merely 50% of anticoagulant-treated patients were given FXa inhibitors, while the rest received LMWH or vitamin K antagonists. Similarly, up to 94% of anticoagulant users in the Nichetti study were administered LMWH. Accordingly, these negative clinical findings may be partially explained by the insufficient capacity of LMWH to reshape the tumor immune microenvironment, instead of denying the rationality of anticoagulation plus ICIs combination strategies.Third, all current relevant evidence originates exclusively from retrospective analyses and lacks verification via prospective randomized controlled trials. Retrospective studies are inevitably confounded by unmeasured covariates, selection bias and incomplete clinical data, making it impossible to confirm definite causal associations. Hence, these negative results need prudent interpretation. Rather than negating the clinical potential of anticoagulant-ICIs combination therapy, these findings should be regarded as solid rationale for further prospective RCT verification.

A recent systematic review by Kött et al. (159) further supports this view: Although preclinical studies have consistently demonstrated a synergistic effect between anticoagulation and ICI therapy, clinical trial results have been inconsistent due to differences in the class of anticoagulant, tumor type, study design, and patient baseline characteristics.The authors emphasize that FXa inhibitors and antiplatelet agents show the greatest potential for synergy, which explains why Haist et al. reported positive findings with FXa inhibitors, whereas Johannet, Nichetti, and others failed to detect any significant differences. In other words, a negative result may not necessarily rule out the potential of anticoagulation combined with ICIs; rather, it suggests the need for more precise drug selection and patient stratification, as well as the need for future prospective studies targeting specific drugs and specific tumor types.

### 6.3 Targeting platelets: dual regulation of effector molecules

Platelets serve as a key interface between the coagulation system and the immune system. Strategies targeting platelets can both directly reverse immunosuppressive TME and repurpose platelets as targeted delivery vehicles to enable precision immunotherapy.

#### 6.3.1 Antiplatelet agents: aspirin

Aspirin suppresses platelet activation via inhibiting the COX-1/TXA_2_ signaling pathway. Yang et al. confirmed in multiple murine tumor models that low-dose aspirin could reverse T cell-mediated immunosuppression by blocking platelet COX-1 and TXA_2_ synthesis, potentiate cytokine functions, and downregulate the expression of exhaustion-related markers including PD-1 and TOX (160). A phase II clinical trial enrolling 122 patients with platinum-resistant ovarian cancer explored the efficacy of atezolizumab combined with bevacizumab ± aspirin. The outcomes revealed that although this triple combination failed to markedly improve 6-month PFS, it exhibited favorable safety profiles without elevating the risk of severe bleeding events, and thus shows promising potential for further clinical optimization (161).

#### 6.3.2 Targeted drug delivery via modified platelets

An even more innovative strategy involves modifying platelets to serve as carriers for tumor-targeted drug delivery. Wang et al. designed the fusion protein tTF-RGD to target tumor neo-vessels, inducing local coagulation to form a scaffold that recruits anti-PD-1 antibody-conjugated platelets.In various tumor models, the combination therapy of tTF-RGD and P-aPD-1 safely induced tumor-specific thrombosis, significantly suppressed tumor growth, and prolonged survival, while increasing the number of CD8^+^ T cells and IFN-γ^+^ Teffs within the tumor (162). Wu and colleagues developed a nanomedicine, cRGD-NP@A, that targets tumor-associated platelets. In models of melanoma, lung cancer, breast cancer, and bladder cancer, this drug disrupts platelet-tumor interactions, reduces TAP-derived TGF-β1 levels, and improves THS. Following combination therapy with anti-PD-1 antibodies, there was increased CD8^+^ T cells infiltration and enhanced function, a reduction in Tregs, and heightened activity of NK cells and DCs. This approach significantly prolonged survival without causing notable toxicities, offering a new direction for low-toxicity, high-efficacy combination immunotherapy (163).

### 6.4 Targeting inflammatory factors and complement: interventions at soluble cross-links

Inflammatory cytokines (such as IL-6) and complement components (such as C5a) serve as both drivers of THS and key maintainers of immunosuppressive TME, acting as soluble crosstalk nodes. Targeting these soluble factors can achieve dual regulation of coagulation and immunity without directly increasing the risk of bleeding, offering certain theoretical advantages.

#### 6.4.1 Drugs targeting IL-6: IL-6 receptor antagonists

IL-6 acts as a critical bridge connecting the coagulation and immune systems, and its signaling pathway is regarded as a highly promising therapeutic target. IL-6 receptor antagonists can block the binding of IL-6 to its receptor, thereby inhibiting downstream signaling activation.Ware et al. verified in pancreatic cancer mouse models that combined administration of IL-6 receptor antagonists and anti-CTLA-4 antibodies exerted markedly stronger tumor growth inhibition than monotherapy. This synergistic efficacy is closely correlated with elevated intratumoral T cell infiltration and enhanced IFN-γ secretion by CD4^+^ T cells. These findings indicate that targeting the IL-6 pathway can effectively remodel the tumor immune microenvironment and potentially reverse immune resistance in cold tumors including pancreatic cancer (164).

#### 6.4.2 Drugs targeting C5aR: C5aR antagonists

The complement system, in particular its effector molecule C5a, plays a central role in the coagulation-immune network. Inhibiting C5a and its receptor C5aR is considered a dual strategy that simultaneously blocks both coagulation activation and immunosuppression (165). In high-grade serous ovarian cancer, high expression of C5AR1 is associated with poor patient prognosis; its inhibitor, PMX53, can inhibit tumor growth, remodel the immune microenvironment, and synergistically enhance the efficacy of anti-PD-1 therapy (166). In gastric cancer, C5AR1 is primarily expressed on TAMs; the C5AR1 antagonist PMX205 can reprogram the macrophage phenotype, attenuate their immunosuppressive function, and activate the cytotoxicity of CD8^+^ T cells. When used in combination with anti-PD-1 therapy, synergistically enhances the expression of effector molecules on CD8^+^ T cells and promotes tumor cells apoptosis (167).

### 6.5 Metabolic interventions

As previously discussed in detail, metabolic products such as lactate, Gln, tryptophan, and adenosine play a key role in driving the hypercoagulable-immunosuppressive vicious cycle. However, clinical evidence supporting the combination of targeted drugs acting on metabolic pathways with ICIs is extremely scarce, which represents the most significant translational gap in this field.

With regard to lactate metabolism, although lactate has been shown to promote THS through multiple mechanisms, there are currently no clinically available selective lactate pathway inhibitors. Although the lactate dehydrogenase (LDH) inhibitor FX-11 has demonstrated antitumor potential in pancreatic cancer and neuroblastoma models, its effects on the coagulation system have not yet been evaluated (168, 169). In the field of tryptophan metabolism, the *IDO1* inhibitor epacadostat has attracted significant attention, as preclinical studies have shown it to be a key metabolic regulator of tumor immune evasion and a potential enhancer of anti-PD-1 immune checkpoint therapy. However, results from a large Phase III clinical trial evaluating its combination with pembrolizumab for the treatment of advanced melanoma indicated that epacadostat provided no additional benefit (170). This failure suggests that highly selective targeting of *IDO1* is insufficient to overcome the tumor’s complex metabolic compensation network, and that tumors can evade immune suppression through bypass mechanisms such as *TDO* and *IDO2*. In the field of adenosine metabolism, CD39/CD73 inhibitors are currently in early-stage clinical trials. Preliminary data indicate that the combination of a CD39 inhibitor and an anti-PD-L1 monoclonal antibody can improve immunosuppressive TME, enhance antitumor immune activity, and achieve a certain degree of disease control; however, existing studies have limited sample sizes and have not yet conducted stratified analyses based on specific immune phenotypes (171). Currently, there are no mature clinical-stage drugs targeting Gln and arginine metabolism. This gap suggests that future efforts should focus on strengthening drug development at the intersection of metabolism, immunity, and coagulation, and exploring strategies that simultaneously target multiple metabolic pathways.

Therapeutic strategies targeting the malignant cycle of “hypercoagulability and immunosuppression” in tumors are shifting from traditional anticoagulation toward the synergistic regulation of coagulation and immunity. Current intervention strategies are gradually forming a comprehensive, tiered system that can target key crossroads at various stages: upstream (TF/PAR), midstream (coagulation factors and platelets), and downstream (inflammatory factors such as IL-6 and the complement system, including C5a). However, interventions targeting metabolic pathways remain relatively limited. These agents not only inhibit coagulation activation and reduce the risk of tumor-associated thrombosis, but also reshape immunosuppressive microenvironment and enhance the efficacy of immunotherapy, thereby providing dual benefits of anticoagulation and immune enhancement. Although there is some heterogeneity and conflicting results regarding efficacy across different clinical studies, the use of prospective randomized controlled trials, stratified analyses by drug class and tumor type, and biomarker-based patient selection strategies will be key to advancing this field from basic research to clinical translation and ultimately improving outcomes for cancer patients.

## 7 Current challenges and future prospects

### 7.1 Safety

Targeted therapies that bidirectionally modulate the hypercoagulable-immune axis currently face key safety challenges: the combined risk of bleeding complications and immune-related adverse events. On the one hand, the combined use of anticoagulants and immunotherapy has significantly increased the incidence of severe bleeding events, such as intracranial hemorrhage and gastrointestinal bleeding. Mild to moderate bleeding, including mucosal bleeding and subcutaneous hematomas, is also particularly common. Such adverse reactions directly reduce patient treatment compliance (172). On the other hand, immunotherapeutic agents are prone to inducing immune-related adverse events, such as rashes and colitis (173). This drug-induced inflammatory damage, combined with the irritating effects of anticoagulants on target organs, further increases the risk of organ injury. Therefore, it is necessary to establish a comprehensive safety management system covering the entire treatment cycle: screen high-risk patients before treatment, dynamically monitor bleeding- and irAE-related indicators during treatment, and adjust treatment regimens in a timely manner, strengthen patient education to facilitate early symptom recognition; and actively develop bleeding risk prediction models to implement personalized safety management by integrating individual patient characteristics.

### 7.2 Mechanistic and clinical challenges

In terms of tumor heterogeneity, as mentioned in the introduction, tumors with a highly procoagulant phenotype exhibit intrinsic resistance to ICIs (174). In contrast, tumors with a hypocoagulable phenotype demonstrate a better response to combination therapy, and high coagulation heterogeneity among tumor types may be one of the key factors determining the success or failure of immunotherapy. Given the significant intratumoral heterogeneity, the relationship between tumor histological characteristics and immune responses varies markedly not only across different types of cancer but also among different subtypes of the same malignancy. Therefore, it is imperative to decipher the specific mechanisms of different cancer types and subtypes and establish patient stratification strategies based on the hypercoagulable phenotype. This requires conducting integrated, pan-cancer cohort studies, using multi-omics analysis to identify the key drivers of the hypercoagulable-immune axis, and establishing a precise “cancer type–target” knowledge base.

At the clinical level, there is currently a lack of specific biomarkers capable of reliably predicting treatment response and safety risks. Although commonly used markers such as prothrombin and D-dimer are of reference value (175–177), their predictive ability is limited, and they cannot provide dynamic risk assessment during treatment. Therefore, there is an urgent need to develop and validate highly specific biomarkers through multicenter clinical trials and establish standardized testing protocols.

In addition, the combination of anticoagulants and immunotherapy poses a challenge due to multidrug resistance in some patients. Whether primary or acquired resistance, both constitute key barriers to the clinical efficacy of this combination therapy, and their molecular mechanisms remain unclear. To address this challenge, whole-exome sequencing and single-cell sequencing should be performed on patients with multidrug resistance. This approach can precisely identify the mutations and pathways driving drug resistance, thereby guiding the development of novel therapeutic drugs to overcome resistance.

### 7.3 Clinical translation and drug development

At the clinical translation stage, there is currently a lack of unified treatment guidelines and robust support from large-scale clinical evidence. Standardized guidelines for key clinical parameters—such as drug selection, dosage, and treatment duration—have yet to be established, and treatment regimens tailored to specific cancer types also require further refinement. In drug development, however, poor druggability remains a key challenge, particularly for bifunctional and multi-target therapeutic agents, where there is still significant room for improvement in physicochemical stability and target specificity. Meanwhile, targeted therapies for metabolic pathways remain extremely limited. In the future, efforts should focus on the following areas: First, develop standardized clinical treatment guidelines based on high-quality clinical trials, promote the optimization of drug structures and the development of novel delivery systems, and improve drug druggability and tissue penetration. Second, conduct large-scale real-world studies to systematically collect clinical data and refine the efficacy and safety evaluation system. Third, prioritize the development of drugs targeting specific metabolic pathways to break the self-reinforcing cycle of metabolic components within the “hypercoagulable-immunosuppressive” vicious cycle.

## 8 Conclusion

In summary, THS and immunosuppressive TME form a mutually reinforcing, self-sustaining vicious cycle, which leads to immunotherapy resistance and an increased risk of thrombosis. THS exacerbates immunosuppression by inhibiting immune cells function via procoagulant factors, inducing hypoxia, and forming physical barriers. Conversely, specific cellular components and factors in the immunosuppressive microenvironment also directly activate the coagulation pathway, drive coagulation-fibrinolysis imbalance, and further consolidate THS through metabolic reprogramming. Currently, therapeutic strategies targeting the coagulation-immune axis have shown great promise, but they remain insufficient in terms of targeted therapies for the metabolic component. Although clinical translation faces numerous challenges, this combination therapy is expected to become a new and effective approach for improving the prognosis of cancer patients.
